# Foot Shape in Women With and Without Hallux Valgus Determined Using 3‐Dimensional Foot Scanning

**DOI:** 10.1002/jfa2.70234

**Published:** 2026-10-21

**Authors:** Gabriel R. Limanou, Polly Q. X. Lim, Merridy J. Lithgow, Andrew K. Buldt, Shannon E. Munteanu, Hylton B. Menz

**Affiliations:** ^1^ Discipline of Podiatry School of Allied Health, Human Services and Sport La Trobe University Melbourne Victoria Australia; ^2^ Faculty of Sports Science and Center of Excellence in Exercise Physiology for Special Populations Chulalongkorn University Bangkok Thailand

**Keywords:** 3‐dimensional, foot scan, foot shape, hallux valgus, women

## Abstract

**Introduction:**

Hallux valgus (HV) is a prevalent forefoot deformity that alters foot shape and occurs more frequently in women and older adults. Few studies have investigated the influence of HV on three‐dimensional (3D) foot structure in age‐matched samples.

**Methods:**

This cross‐sectional study utilised data from two separate studies conducted in Melbourne, Australia. Twenty women (mean ± SD age = 63.8 ± 8.7 years) with HV were age‐matched to 20 women without HV (63.8 ± 8.6 years). Participants underwent 3D foot scanning, comparing measurements of the most symptomatic HV feet with corresponding control feet. Differences in foot dimensions between groups were calculated using independent sample *t‐*tests.

**Results:**

Women with HV demonstrated larger foot breadth (103.1 ± 6.2 vs. 98.0 ± 5.6, mean difference [MD] = 5.13 mm, *p* = 0.009), ball girth circumference (245.9 ± 13.9 vs. 234.1 ± 11.5, MD = 11.86 mm, *p* = 0.006), heel breadth (65.9 ± 3.9 vs. 62.8 ± 3.8, MD = 3.13 mm, *p* = 0.015), height of top of ball girth (39.9 ± 2.8 vs. 37.4 ± 2.2, MD = 2.56 mm, *p* = 0.003), and toe 1 angle (24.1 ± 8.0 vs. 9.8 ± 8.0, MD = 14.29°, *p* < 0.001). There were no differences in rearfoot position (as indicated by heel bone angle) observed between the groups (3.0 ± 2.8 vs. 3.1 ± 3.0°, *p* = 0.952).

**Conclusion:**

Women with HV exhibited a broader forefoot and heel compared with those without the deformity. Moreover, women with HV displayed a larger ball girth circumference and height of the top of the ball girth, suggesting that vertical dimensional changes occur in this condition. These findings suggest that individuals with HV may benefit from adequate footwear fitting and new lacing techniques and inform the development of targeted footwear modifications or HV‐specific footwear manufacturing guidelines.

## 1 Introduction

Hallux valgus (HV) is a common forefoot condition that impacts overall foot shape and mobility. It is characterised by lateral deviation of the hallux and/or medial deviation of the first metatarsal head [1, 2]. HV affects approximately 30% of women, twice the rate observed in men, and its prevalence increases with age, affecting one in three individuals over 65 years [3]. After the age of 50, one in three individuals with HV demonstrates worsening alignment of the first metatarsophalangeal joint, indicating that the condition is progressive [4].

HV is associated with foot pain [5], impaired physical function [6], depression [7], and reduced general and foot‐specific health‐related quality of life [8]. HV also compromises balance, increasing fall risk [9]. Difficulty in obtaining properly fitting footwear remains a commonly reported issue, particularly in relation to adequate width [10, 11]. Moreover, HV poses a significant economic burden, contributing to over 60,000 general practice visits annually in Australia, costing approximately AUD$2.2M in Medicare subsidies for initial consultations [12], and around 5000 surgical procedures by orthopaedic surgeons each year, adding AUD$2M in operative costs [13].

Interventions for HV focus on pain relief and slowing the progression of the deformity. Therefore, conservative and surgical management have been recommended [14, 15]. Non‐surgical approaches are considered in the initial management of HV [14]. Conservative management comprises footwear advice or modification, custom and prefabricated orthotic devices, HV padding, and various strengthening programs [16]. A constrictive toe box is a recognised risk factor for HV, so wider‐fitting footwear is commonly recommended [17, 18].

Surgical interventions for HV are indicated for those with persistent pain or inflammation and for patients who require structural realignment [19]. Limited footwear selection following surgery is a common concern [11, 19]. Indicators of surgical success extend beyond radiographic correction and include patient‐reported outcomes such as pain reduction, improved foot function, and greater satisfaction with the range of footwear selection [11]. As such, employing a patient‐centred approach that incorporates both objective correction and subjective quality of life improvements is essential for optimising surgical outcomes in the management of HV.

Understanding how foot shape changes with HV is important, given that altered foot shape contributes to pain, and difficulty finding comfortable footwear can also influence surgical intervention decisions. Most HV‐related studies utilise conventional linear radiographic assessments to focus on angular measurements such as the HV angle and intermetatarsal angle [2, 20]. More recently, weightbearing three‐dimensional (3D) computed tomography scans have been used to provide greater insight into osseous alignment [21, 22]. While useful for surgical planning, these measurements do not capture the influence of HV on the overall foot surface contour and are limited due to high cost and radiation exposure. In contrast, 3D foot scanning provides a non‐ionising method for quantifying external foot morphology and surface dimensions [23].

Very few studies have investigated the influence of HV on 3D foot structure. Based on 3D foot scans of 37 older participants with HV compared with 275 participants without the deformity, Mickle et al. reported increased ball girth and width, medial and lateral ball lengths, heel bone and first toe angle, as well as a reduction in ball height, instep height, and navicular height in those with HV [24]. A more recent study involving a large‐scale analysis of 959,074 scans obtained across three continents demonstrated that there was a moderate association between HV and wider ball width [2]. However, in this study, limited foot shape parameters were measured and scans were taken barefoot and in socks due to constraints imposed by the fast‐paced retail environment in which the scans were collected. Furthermore, the study did not use markers on key bony landmarks during the scanning, which is unlike conventional 3D scanning methods. Importantly, neither study matched participants according to age, potentially introducing age‐related confounding effects.

Therefore, the aim of this study was to compare the 3D foot shape of women with and without HV, matched for age. Findings from this study may inform future practices on footwear design, lacing techniques, and other modifications to accommodate HV‐related structural deformities.

## 2 Methods

### 2.1 Study Design

This cross‐sectional, observational study compared a range of foot dimensions obtained from 3D scans between women with and without HV, matched for age. The study is reported according to the recommendations of the Strengthening the Reporting of Observational Studies in Epidemiology Statement (STROBE) [25] (Table S1), as well as the 16‐item Consistent ReporTing Three‐dimensional sCanning (CRITIC) checklist (Table S2) [23]. Data were extracted from two separate studies, each of which recruited a distinct population group incorporated into the current study. Informed consent was provided by all participants from each study.

### 2.2 Participants

Participants were recruited via convenience sampling. Participants with HV were recruited as part of a feasibility trial prior to any intervention (ethics number HEC20474), and participants without HV were recruited as part of a larger database study on 3D foot scanning (ethics number HEC19538). Participants residing in Melbourne, Victoria, Australia were recruited via postal invitation from a database of patients who had previously received podiatry care at the La Trobe University Podiatry Clinic. Further, invitations were distributed to staff members from La Trobe University, along with social media advertisements and distribution of posters to attract potential volunteers.

Both studies incorporated strict inclusion and exclusion criteria. For participants with HV, the inclusion criteria were: (i) aged 40 years and above, (ii) women (self‐identified), (iii) experiencing pain (rated ≥ 3 on an 11‐point numerical rating scale) at the first metatarsophalangeal joint for at least 12 weeks, (iv) ability to walk > 50 m unaided, (v) proficiency with the English language and (vi) at least moderate HV on their most severely affected foot assessed using the Manchester scale [26]. The exclusion criteria were: (i) history of surgical intervention on either hallux, (ii) lower limb or partial foot amputation, (iii) systemic inflammatory condition(s), (iv) neurological disease that interferes with walking, (v) having worn arch contouring foot orthoses in the past 12 weeks, (vi) performed foot exercises in the past 12 weeks, or (vii) possessing an injury of the lower limb or back that may have interfered with reaching their feet.

For non‐HV participants, the inclusion criteria were: (i) aged 18 years and above, and (ii) not experiencing foot pain. The exclusion criteria were: (i) history of suspected or diagnosed systemic inflammatory joint conditions, (ii) diagnosed neuropathy, (iii) prior surgery to any musculoskeletal structures in the foot, or (iv) fractures in the foot. Participants who self‐identified as women were age‐matched to those with HV.

### 2.3 Sample Size Calculation

The sample size was determined by the availability of participants with HV, as these were recruited from a pilot and feasibility trial. As a general rule of thumb, pilot studies are recommended to recruit a minimum of 12 participants per group [27]. Therefore, to anticipate a 15% drop‐out rate, 14 participants were recruited for each intervention group in the pilot and feasibility trial, resulting in a total of 28 HV participants [28]. Among the 28 available participants in the pilot and feasibility study, 20 were able to be age‐matched (± 5‐year tolerance) with the non‐HV participants in the database study, resulting in an overall sample size of 40 (20 participants with HV and 20 non‐HV) for the current study. Using G*Power, a sample size of 20 per group was deemed sufficient to identify a large effect size (Cohen's *d ≥* 0.80 [29]) with a power of 0.80 when independent sample *t*‐tests are utilised [30].

### 2.4 Protocol

Data collection occurred between July 2021 and January 2023. Participants completed a structured, self‐report questionnaire to obtain information regarding their prior medical history and medications. Age was self‐reported in years, height was measured using a stadiometer in centimetres, weight was measured using scales in kilograms, and body mass index (BMI; kg/m^2^) was calculated. Cultural background (including ethnicity) and education level were assessed. Ethnicity was assessed using the Australian Standard Classification of Cultural and Ethnic Groups, which has a three level hierarchical structure consisting of broad groups, narrow groups, and cultural and ethnic groups [31].

### 2.5 Foot Shape Measurement

Foot shape of participants was measured using a 3D INFOOT scanner high type, IFU‐S‐01 (I‐Ware Laboratory Co. Ltd, Osaka, Japan). The scanner uses 4 laser projectors and 8 charge‐coupled device cameras to capture a variety of foot dimensions consistent with the Institute of Electrical and Electronics Engineers (IEEE) White Paper [32]. The INFOOT scanner has a resolution of 0.1 mm and scans at 30 mm/second (total capture duration approximately 10 seconds). Six velvet markers with a diameter of 5 mm and a thickness of 2 mm were positioned on anatomical landmarks, providing detailed information available for inspection and quantification to replicate accurate foot morphology [23] (Figure 1). Prior to measurement, the scanner was calibrated according to the manufacturer's instructions. The scanner has previously demonstrated excellent validity relative to radiographic measures and high test‐retest reliability [33, 34]. Foot measurements were obtained under barefoot weightbearing conditions using a single scan. Both feet were scanned; for HV participants, the most severely affected foot was used, and this was compared with the corresponding foot of non‐HV participants. Typical examples of HV and non‐HV scans are shown in Figure 2. Scan measurement data were exported from the INFOOT Measure software to Microsoft Excel (Redmond, Washington, USA).

**FIGURE 1:**
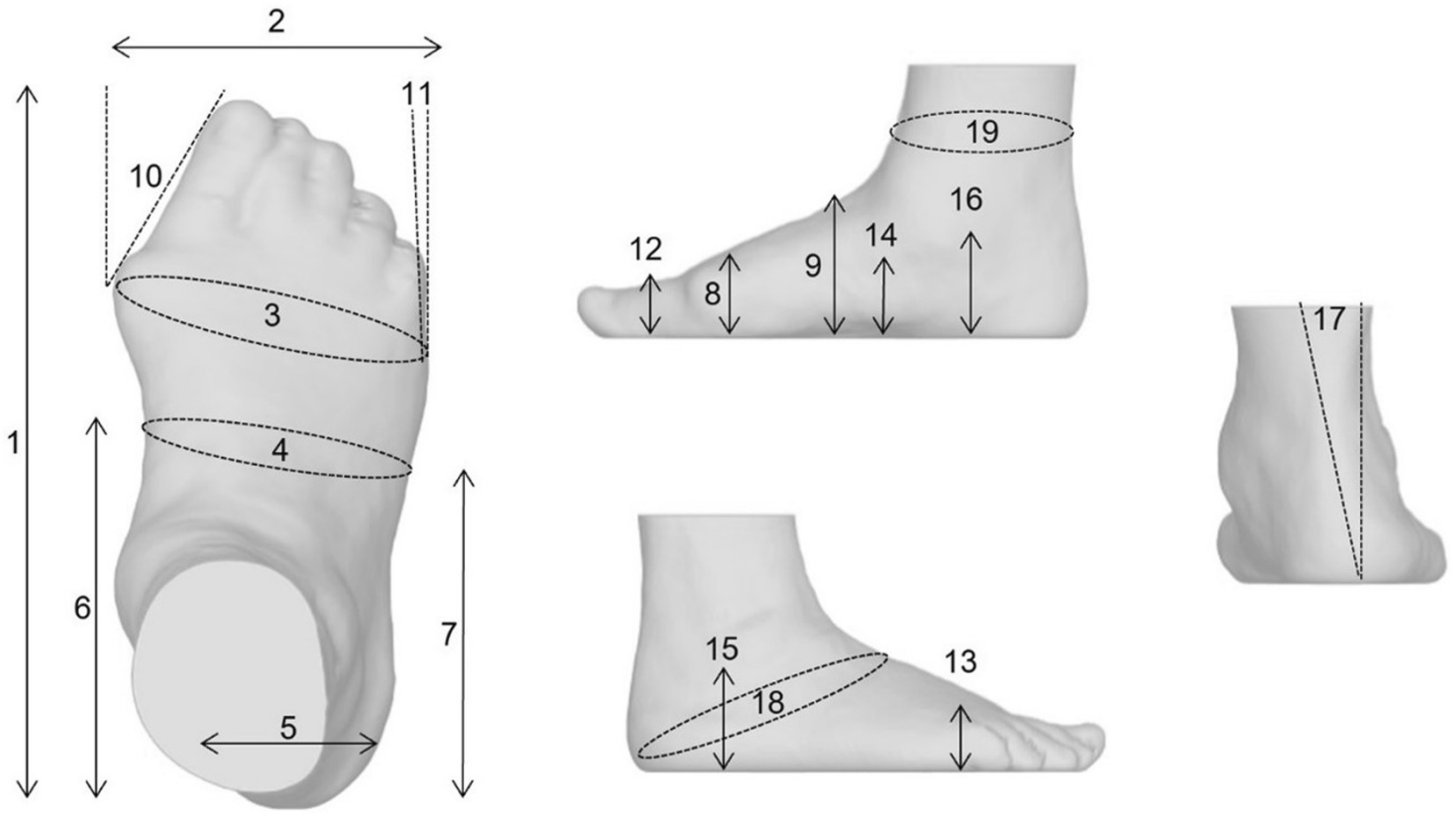
3D foot scan measurements. 1: foot length, 2: foot breadth, 3: ball girth circumference, 4: instep circumference, 5: heel breadth, 6: instep length, 7: fibular instep length, 8: height of top of ball girth, 9: height of instep, 10: toe 1 angle, 11: toe 5 angle, 12: toe 1 height, 13: toe 5 height, 14: height of navicular, 15: height of most lateral point of lateral malleolus, 16: height of most medial point of medial malleolus, 17: angle of heel bone, 18: heel girth circumference, 19: horizontal ankle circumference.

**FIGURE 2:**
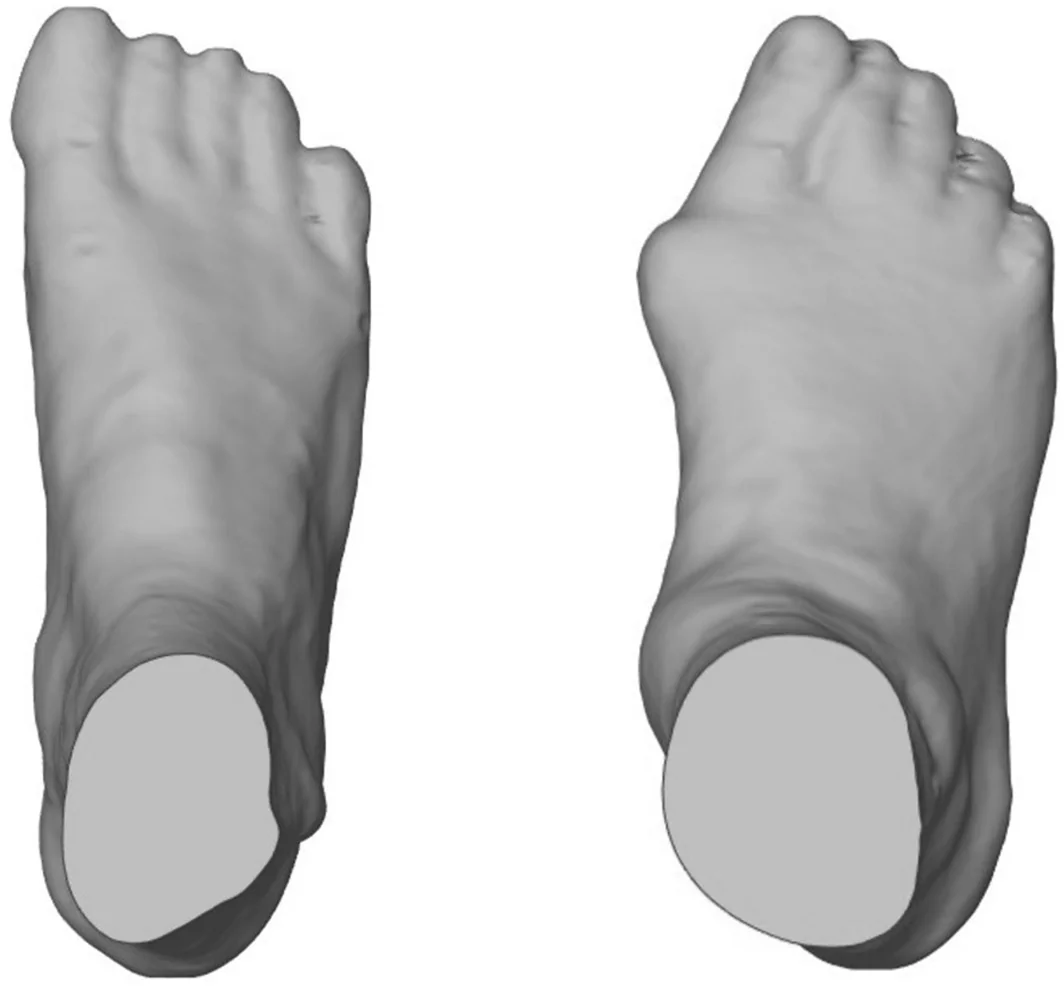
Example 3D foot scan of a participant without (left) and with (right) hallux valgus.

There were 19 foot dimensions measured in this study, these included:

- *foot length*, defined as the pternion (the most posterior point of the heel of the foot) to the farthest point from pternion (measured in millimetres);
- *foot breadth*, defined as the width of the most medial point of ball girth to the most lateral point of ball girth (measured in millimetres);
- *ball girth circumference*, defined as the circumference of a line connecting the most medial point of ball girth to the most lateral point of ball girth (measured in millimetres);
- *instep circumference*, defined as the circumference of a line connecting the highest point of the instep with the ground (measured in millimetres);
- *heel breadth*, defined as the medial to lateral width of the heel region at 16% of foot length and 40 mm from the supporting surface (measured in millimetres);
- *instep length*, defined as the length of the pternion to the most medial point of the ball girth (measured in millimetres);
- *fibular instep length*, defined as the length of the pternion to the most lateral point of the ball girth (measured in millimetres);
- *height of top of ball girth*, defined as the length from the ground to the highest point of ball girth circumference (measured in millimetres);
- *height of instep*, defined as the highest point of the foot at 50% of foot length (measured in millimetres);
- *toe 1 angle*, defined as the angle formed by the medial aspect of the hallux relative to the medial axis of the foot (measured in degrees, with larger values representing greater HV angle);
- *toe 5 angle*, defined as the angle formed by the lateral aspect of the fifth toe relative to the lateral axis of the foot (measured in degrees, with larger values representing greater fifth metatarsophalangeal joint adduction to the midline of the foot);
- *toe 1 height*, defined as the highest point of the hallux relative to the supporting surface (measured in millimetres);
- *toe 5 height*, defined as the highest point of the fifth toe relative to the supporting surface (measured in millimetres);
- *height of navicular*, defined as the height of the navicular relative to the supporting surface (measured in millimetres);
- *height of most lateral point of lateral malleolus*, defined as the height of the lateral malleolus relative to the supporting surface (measured in millimetres);
- *height of most medial point of medial malleolus*, defined as the height of the medial malleolus relative to the supporting surface (measured in millimetres);
- *angle of heel bone*, defined as the angle formed by a line connecting the upper point of the heel angle to the lower point of the heel angle and a vertical line (measured in degrees, with larger values representing greater eversion);
- *heel girth circumference*, defined as the circumference of a line connecting the landing point (the most inferior point of the heel) to the junction point (the point at which the foot becomes the leg) (measured in millimetres);
- *horizontal ankle circumference*, defined as the circumference of a horizontal line at the level of the junction point (measured in millimetres).

### 2.6 Statistical Analysis

Statistical analysis was conducted using IBM SPSS Statistics version 29.0 (IBM Corp, NY, USA). Nominal data (e.g., ethnicity, education, major medical conditions) were reported as frequencies (percentages). All continuous data were explored for normality using a combination of graphical outputs (histograms, box plots, P‐P plots, and Q‐Q plots) and statistical tests (Shapiro‐Wilk, Kolmogorov‐Smirnov, skewness, and kurtosis). Extreme outliers (defined as values extending beyond the third interquartile range of the standard deviation) were removed from the analysis to minimise distortion of summary statistics and ensure the validity of parametric assumptions. Independent sample *t*‐tests were conducted to compare differences in foot parameters between age‐matched participants with and without HV. In all analyses, *p* < 0.05 was considered statistically significant. Effect sizes were determined by calculating Cohen's *d*, defined as the mean difference between the two groups divided by the pooled standard deviation (SD) [35]. The following interpretation of Cohen's *d* was used: < 0.1 = tiny, 0.1–0.2 = very small, 0.2–0.5 = small, 0.5–0.8 = medium, 0.8–1.2 = large, 1.2–2 = very large, and *d* > 2 = huge [29]. We also performed a sensitivity analysis adjusting for BMI, as this was not matched between the groups.

## 3 Results

### 3.1 Participant Characteristics

A total of 40 participants (20 participants in each group, HV and non‐HV) were age‐matched and included. Participant characteristics are shown in Table 1. Both the HV and non‐HV groups exhibited a mean age of 63.8 years, with a SD of 8.7 and 8.6, respectively. The groups had a similar average BMI (SD) of 27.5 (4.3) kg/m^2^ (HV participants) and 26.0 (5.2) kg/m^2^ (non‐HV participants). Participants from both groups most commonly had Oceanian ethnicity (40% in the HV group and 70% in the non‐HV group). Both groups displayed a range of medical conditions, with osteoarthritis as the most frequently self‐reported comorbidity in HV participants (45%).

**TABLE 1:** Participant characteristics in participants without and with hallux valgus (HV).

	No HV (*n* = 20)	HV (*n* = 20)
Age—mean (SD), years	63.8 (8.6)	63.8 (8.7)
Height—mean (SD), cm	162.4 (9.0)	160.0 (6.9)
Weight—mean (SD), kg	68.8 (15.8)	70.5 (12.1)
Body mass index—mean (SD), kg/m^2^	26.0 (5.2)	27.5 (4.3)
Ethnicity		
Oceanian	14 (70)	8 (40)
South‐East Asian	4 (20)	2 (10)
Southern European	1 (5)	3 (15)
United Kingdom	1 (5)	1 (5)
Anglo‐Indian	0 (0)	2 (10)
North African	0 (0)	4 (20)
Highest schooling		
Primary	3 (15)	1 (5)
High school	4 (20)	6 (30)
College/TAFE^a^	10 (50)	4 (20)
University	0 (0)	4 (20)
Postgraduate	3 (15)	5 (25)
Major medical conditions		
Hearing problems	4 (20)	1 (5)
Leg cramps	2 (10)	5 (25)
Diabetes	1 (5)	0 (0)
Stroke	0 (0)	0 (0)
Heart disease	0 (0)	1 (5)
Hypertension	4 (20)	3 (15)
Osteoarthritis	5 (25)	9 (45)
Fracture	1 (5)	3 (15)

*Note:* Values are *n* (%) unless otherwise noted.

^a^ TAFE = technical and further education (trade qualification).

### 3.2 Foot Shape Differences

Table 2 presents foot shape parameters (mean, SD) for both groups as well as findings of statistical analyses for differences between groups. One extreme outlier for the height of the most lateral point of the lateral malleolus was excluded from this analysis. Participants with HV demonstrated increased foot breadth (103.1 ± 6.2 vs. 98.0 ± 5.6, MD = 5.13 mm, 95% CI 1.34–8.92, *p* = 0.009), ball girth circumference (245.9 ± 13.9 vs. 234.1 ± 11.5, MD = 11.86 mm, 95% CI 3.68–20.03, *p* = 0.006), heel breadth (65.9 ± 3.9 vs. 62.8 ± 3.8, MD = 3.13 mm, 95% CI 0.66–5.60, *p* = 0.015), height of top of ball girth (39.9 ± 2.8 vs. 37.4 ± 2.2, MD = 2.56 mm, 95% CI 0.92–4.19, *p* = 0.003), and toe 1 angle (24.1 ± 8.0 vs. 9.8 ± 8.0, MD = 14.29°, 95% CI 9.19–19.39, *p* < 0.001) compared with participants without HV. Effect sizes were large to very large for these comparisons (Cohen's *d* ranging from 0.83 to 1.83) and were statistically significant at *p* < 0.05. A sensitivity analysis adjusting for BMI differences between groups slightly attenuated these differences, but the overall pattern of findings was unchanged.

**TABLE 2:** 3D foot shape parameters in participants without and with hallux valgus (HV).

Parameter	No HV (*n* = 20)	HV (*n* = 20)	Mean difference (95% CI)	*d*	Effect size	% difference	p‐value	Adjusted *p*‐value^b^
Foot length	243.2 (13.7)	242.2 (14.9)	−0.95 (−10.11 to 8.22)	0.07	Tiny	0	0.836	0.747
Foot breadth	98.0 (5.6)	103.1 (6.2)	5.13 (1.34–8.92)	0.89	Large	5	0.009	0.011
Ball girth circumference	234.1 (11.5)	245.9 (13.9)	11.86 (3.68–20.03)	0.95	Large	5	0.006	0.016
Instep circumference	231.4 (11.0)	237.5 (15.0)	6.17 (−2.26 to 14.60)	0.48	Small	3	0.147	0.248
Heel breadth	62.8 (3.8)	65.9 (3.9)	3.13 (0.66–5.60)	0.83	Large	5	0.015	0.027
Instep length	181.3 (10.7)	178.0 (11.8)	−3.34 (−10.53 to 3.86)	0.30	Small	2	0.353	0.319
Fibular instep length	158.4 (10.5)	155.9 (11.5)	−2.55 (−9.59 to 4.50)	0.23	Small	2	0.469	0.364
Height of top of ball girth	37.4 (2.2)	39.9 (2.8)	2.56 (0.9–4.19)	1.02	Large	6	0.003	0.005
Height of instep	64.2 (4.5)	63.2 (4.9)	−0.98 (−4.00 to 2.04)	0.22	Small	2	0.516	0.427
Toe 1 angle (°)	9.8 (8.0)	24.1 (8.0)	14.29 (9.19–19.39)	1.83	Very large	146	< 0.001	< 0.001
Toe 5 angle (°)	11.9 (5.2)	11.6 (5.4)	−0.30 (−3.69 to 3.10)	0.06	Tiny	−3	0.861	0.751
Toe 1 height	21.6 (2.3)	22.5 (2.4)	0.98 (−0.53 to 2.49)	0.39	Small	4	0.197	0.230
Toe 5 height	17.1 (3.0)	17.6 (4.3)	0.51 (−1.85 to 2.87)	0.14	Very small	3	0.665	0.751
Height of navicular	40.7 (6.7)	43.3 (6.9)	2.59 (−1.76 to 6.92)	0.39	Small	6	0.235	0.178
Height of most lateral point of lateral malleolus^a^	66.4 (5.6)	70.1 (6.4)	3.73 (−0.16 to 7.61)	0.63	Medium	5	0.059	0.035
Height of most medial point of medial malleolus	77.7 (6.9)	78.2 (6.3)	0.47 (−3.77 to 4.70)	0.08	Tiny	1	0.825	0.719
Angle of heel bone (°)	3.1 (3.0)	3.0 (2.8)	−0.06 (−1.89 to 1.78)	0.04	Tiny	−3	0.952	0.925
Heel girth circumference	311.0 (17.3)	318.7 (18.4)	7.68 (−3.74 to 19.10)	0.44	Small	2	0.181	0.299
Horizontal ankle circumference	250.8 (15.5)	253.1 (17.9)	2.26 (−8.45 to 12.97)	0.14	Very small	1	0.672	0.930

*Note:* Cohen's *d* interpretation is as follows: < 0.1 = tiny, 0.1 to 0.2 = very small, 0.2 to 0.5 = small, 0.5 to 0.8 = medium, 0.8 to 1.2 = large, 1.2 to 2 = very large, and *d* > 2 = huge. Values are mean (SD) millimetres unless otherwise noted.

^a^ one outlier deleted.

^b^ Adjusted for BMI.

## 4 Discussion

The aim of this study was to compare foot shape between women with and without HV, matched for age. Participants with HV demonstrated increased foot breadth, ball girth circumference, heel breadth, height of top of ball girth, and toe 1 angle compared with participants without HV. Effect sizes ranged from large to very large for these comparisons. Our findings indicate that women with HV have a broader foot shape and a higher top of ball girth, demonstrating a significant difference in transverse and vertical dimensions of the foot between those with and without the deformity.

Despite the high prevalence of HV in women, there are very few studies comparing foot dimensions in individuals with and without HV using a 3D scanner [2, 24]. Similar to our study, participants in previous studies demonstrated large toe 1 angles, indicating that individuals with moderate to severe HV were included [2, 24]. As expected, the 1 toe angle was significantly greater in participants with HV than in non‐HV participants. Our results indicate that women with HV display a broader foot shape, which is in agreement with the findings of previous studies [2, 24]. Additionally, one study documented increased ball width and ball girth circumference in HV participants, consistent with our findings [24].

A previous study proposed that a flatter or more pronated foot type may be associated with moderate‐severe HV [24]. In a previous study, women with HV demonstrated lower navicular height, greater heel bone angle (indicative of greater frontal plane eversion), and reduced instep height [24]. Lower navicular and instep heights, and a greater heel bone angle are indicative of a lower medial longitudinal arch, suggesting a pronated foot posture [24, 36]. These results contrast with our findings, which showed no significant differences in navicular height, heel bone angle, and instep height between women with and without HV. Therefore, the relationship between foot posture and HV remains inconclusive, with different studies reporting inconsistent findings [24, 37]. This may be at least partly due to sample size and age differences in participants.

Our study revealed a significant difference in heel breadth between the participants with and without HV—this is a novel finding that contrasts with previous work [24] and may be attributed to sex‐specific anatomical variations [38] and the presence of pre‐existing conditions among participants of the previous study. Interestingly, a CT scan study of older women found that those with larger HV were more likely to have a more medially oriented talus and calcaneus [39]. Although these findings do not directly explain the greater heel breadth observed in our study, they suggest that HV‐related morphology may extend beyond the forefoot. Given our small sample and the number of comparisons performed, the heel‐breadth finding should be interpreted cautiously and requires replication [40].

As this cross‐sectional study compared foot dimensions between women with and without HV at a single point in time, we could not determine the temporal relationship of these observations. However, the differences in foot measurements observed in the current study may be explained by several physiological and biomechanical changes that have occurred over time. The wider foot breadth and larger ball girth circumference in women with HV could be attributable to pathological changes involving the first metatarsophalangeal joint, including the formation of medial osteophytes [41], bursal inflammation, and capsular hypertrophy [42]. These changes may also contribute to the increased height of the top of ball girth observed in women with HV. An unexpected finding of this study was the wider heel breadth observed in the HV population. While this difference is not easily explained, it may be due to redistribution of plantar padding or changes in the morphology of the calcaneus associated with the condition.

The broader foot profile observed in people with HV may limit choices when selecting footwear. Individuals with HV often struggle to find shoes that accommodate the enlarged forefoot, leading to discomfort, restricted footwear choices, and reduced quality of life [10]. Footwear compatibility is a major predictor of postoperative satisfaction. One of the measurable outcomes of successful surgery is a reduction in forefoot width. Techniques such as the Chevron and Scarf osteotomies have demonstrated significant decreases in forefoot width [43, 44]. While pain relief and functional improvement are critical, the ability to wear a broader range of shoes—especially for women—is often cited as a key determinant of perceived surgical success. Patients who returned to wearing preferred footwear post‐surgery reported higher satisfaction scores and better overall quality‐of‐life outcomes [11]. This is particularly relevant given that many patients seek surgical intervention not solely for pain, but for the limitations imposed by footwear incompatibility.

The findings from this study offer insights for both clinical practice and footwear design for people with HV. Conservative management of HV typically involves footwear advice and modifications [14, 16]. As such, identifying changes in specific foot dimensions, including increased ball width, ball girth circumference, and heel breadth, may inform the development of anatomically accommodative footwear. In addition, given that people with HV display a higher top of ball girth, which may compromise shoe fit around the midfoot, people with HV may incorporate appropriate lacing techniques to accommodate this prominent region of the foot [45].

This study has several strengths. A major strength of this study is the recruitment of age‐matched samples, which helped minimise the risk of age‐related confounding influencing the observed group differences. The decision to focus solely on women acknowledges sex‐based structural differences in foot morphology [38] and the higher prevalence of HV in women [3, 46, 47]. Additionally, the use of a validated 3D measurement tool enabled detailed and comprehensive measurement of foot dimensions.

However, this study has limitations. First, the 3D scanning tool is restricted to measuring surface shape and therefore cannot delineate the contribution of soft tissue and osseous structures to the measurements. Second, grouping all HV cases together without stratifying by HV severity may obscure fundamental differences in foot parameters across deformity stages. Third, ethnic variability among participants, while enhancing generalisability, introduces potential confounding, as certain ethnic groups (e.g., those from Asia) may be predisposed to more severe HV angles [2]. Fourth, our sample size was relatively small, was conducted on a specific population, and was not specifically powered for the number of foot shape comparisons, so the findings need to be interpreted as preliminary observations only. Lastly, the cross‐sectional study design does not allow for causal inference between foot shape and HV.

Future studies should aim to address these limitations. First, the use of imaging modalities that allow for the simultaneous assessment of soft‐tissue and osseous structures would facilitate a better understanding of the relative contribution of foot shape in HV. Additionally, longitudinal studies stratifying HV severity with the Manchester scale [26] could provide targeted insights into how severity may influence prognosis and biomechanical outcomes. Interestingly, large‐cohort statistical shape‐modelling studies of the hip have demonstrated the ability to identify morphological subgroups that may not be apparent using simpler dimensional measures [48]. Similarly, larger samples may facilitate the investigation of potential HV phenotypes. Moreover, future research on HV should aim to broaden the demographic scope of study populations. Our study predominantly included participants of Oceanian descent, limiting its generalisability to the broader population. Incorporating diverse ethnic groups may uncover cultural or hereditary factors influencing the condition. Expanding sample sizes across age groups and geographical regions may enhance statistical power and improve the reliability of conclusions drawn from future studies. As this study only included women, further studies investigating men with HV would promote a better understanding of sex‐ and gender‐specific differences in HV presentation, progression, and treatment outcomes. Lastly, future studies should consider using a longitudinal approach to explore the causal relationship between foot shape changes and the development of HV.

## 5 Conclusion

Women with HV exhibit a broader forefoot and heel compared with those without the deformity, as well as a larger ball girth circumference and height of the top of the ball girth. These findings highlight that HV is associated with changes in foot shape in both transverse and vertical dimensions. These findings have practical implications in relation to footwear fitting and lacing techniques, and may inform the development of targeted footwear modifications and HV‐specific footwear manufacturing guidelines to accommodate the structural needs of this population.

## Author Contributions

**Gabriel R. Limanou:** formal analysis, writing – original draft, writing – review and editing. **Polly Q. X. Lim:** conceptualization, methodology, data curation, writing – review and editing. **Merridy J. Lithgow:** conceptualization, methodology, data curation, writing – review and editing. **Andrew K. Buldt:** conceptualization, methodology, supervision, writing – review and editing. **Shannon E. Munteanu:** conceptualization, funding acquisition, methodology, supervision, writing – review and editing. **Hylton B. Menz:** conceptualization, funding acquisition, methodology, supervision, formal analysis, writing – review and editing.

## Funding

This study was funded by the La Trobe University Research Focus Area in Sport, Exercise and Rehabilitation Grant‐ready Scheme. The funders were not involved in the design of the study, in the writing of the protocol, or in the decision to submit the manuscript for publication.

## Ethics Statement

This study was approved by the Ethics Committee of La Trobe University (HEC20474 and HEC19538).

## Consent

We obtained written informed consent from all participants in this study.

## Conflicts of Interest

A.K.B. is currently the Co‐Editor‐in‐Chief and H.B.M. is the Emeritus Editor of *Journal of Foot and Ankle Research*. It is journal policy that editors are removed from the decision‐making process of papers they have authored. None of the other authors have competing interests to declare.

## Supporting information


**Table S1:** STROBE statement—checklist of items that should be included in reports of observational studies.

**Table S2:** CRITIC 3D scanning checklist.

## Data Availability

The data that support the findings of this study are available from the corresponding author upon reasonable request.
