# Supplementary material for: Foot Shape in Women With and Without Hallux Valgus Determined Using 3‐Dimensional Foot Scanning
Source: J Foot Ankle Res. 2026 Oct 21;19(4):e70234. doi: 10.1002/jfa2.70234 (PMC13687573; doi:10.1002/jfa2.70234)
Supplement: Supplementary file 1 [file JFA2-19-e70234-s001.docx]

| CRITIC 3D scanning checklist. | | | | |
| --- | --- | --- | --- | --- |
| *No* | *Criteria* | *Item description* | 🗹 | *Page* |
| Participants |  |  |  |  |
| 1 | Sample size | Reported or cited in manuscript | 🗹 | 10 |
| 2 | Men/women | Reported or cited in manuscript | 🗹 | 10 |
| Equipment | | | |  |
| 3 | Scanner model | Scanner name/model | 🗹 | 7 |
| 4 | Scanner type | Laser, structured light etc. | 🗹 | 7 |
| 5 | Scanner resolution |  | 🗹 | 7 |
| 6 | Scanner accuracy |  | 🗹 | 7 |
| 7 | Scanner capture duration (secs) |  | 🗹 | 7 |
| 8 | Scanner calibration methods | Reported or cited in manuscript | 🗹 | 8 |
| 9 | Scanner reliability | Reported or cited in manuscript | 🗹 | 8 |
| 10 | Markers used | Number, placement - automated or manual or both | 🗹 | 7 |
| 11 | Number of scans | Number of scans reported per foot | 🗹 | 8 |
| Participant condition | | | |  |
| 12 | Weightbearing | According to IEEE standards – bipedal, unipedal, partial/semi weightbearing, non-weightbearing, external bodyweight | 🗹 | 8 |
| 13 | Barefoot/socks |  | 🗹 | 8 |
| 14 | Unilateral/bilateral scanning | Justification provided if unilateral scanning performed | 🗹 | 8 |
| Measurements | | | |  |
| 15 | Foot measures used | Definitions according to IEEE standards | 🗹 | 7 |
| Post processing | | | |  |
| 16 | Software used | Type, Automated/manual/both – please specify in comments | 🗹 | Automated |
